# Socio-Cognitive Processes and Peer-Network Influences in Defending and Bystanding

**DOI:** 10.1007/s10964-022-01643-z

**Published:** 2022-07-08

**Authors:** J. Ashwin Rambaran, Tiziana Pozzoli, Gianluca Gini

**Affiliations:** 1 Interdisciplinary Social Science (ISS) and European Research Centre on Migration and Ethnic Relations (Ercomer), Utrecht University, Utrecht, The Netherlands; 2 School of Education (SoE) and Combined Program in Education and Psychology (CPEP), University of Michigan, Ann Arbor, MI USA; 3 Department of Developmental Psychology and Socialization, University of Padua, Padua, Italy

**Keywords:** Peer defending, Passive bystanding, Self-efficacy, Moral distress, Socialization

## Abstract

Peers are critical to defending and bystanding during episodes of bullying. This study investigates the extent to which friends can shape defending and bystanding as well as social cognitions associated with these two behaviors (i.e., perceptions of self-efficacy and moral distress). The study sample consisted of *n* = 1354 early and middle adolescents (7th‒10th grade; 81.4% Italian; 51.3% boys) in northern Italy. Employing a longitudinal social network analytic approach, using stochastic actor-oriented modeling, this study found that adolescents become more similar or stay similar to their friends in both behaviors and perceptions, with no clear indication that students select friends based on similar levels of behaviors or perceptions. The findings illustrate how defending and bystanding behaviors and related social cognitions are developed within friend (peer) networks.

## Introduction

While most bystanders recognize that bullying is wrong and that they would like to help the victims, at the same time many victims remain nevertheless undefended (Salmivalli, 2010). This suggests the presence of a defending-bystanding paradox that is not well understood: Why do some defend, while others do not? Research suggests that peers, and friends, in particular, are critical to defending and bystanding choices as peers are present in most bullying situations (Salmivalli, 2010). Yet, limited attention has been given to the critical role that certain socio-cognitive processes play in the defending-bystanding process, such as personal beliefs about how well students are able to support the victims (Gini, Pozzoli et al., 2022) or negative emotional experiences when they are unable to support them (Gini et al., 2020). Socio-cognitive processes of defending and bystanding are seldom examined by themselves and never in conjunction with peer-group processes. Extant literature on social cognitions of defending and bystanding during adolescence has almost exclusively examined its effects on defending and bystanding, with very few exceptions (Pozzoli and Gini, 2013). Nonetheless, theory suggests that not only are social cognitions related to the self, but also to socialization processes (Bandura, 1977), which in the context of defending and bystanding could be sharing of information about one’s (in)abilities concerning defending of victims within the peer group. To address this gap in the literature, this study examines the relation between socialization and (social cognitions of) defending and bystanding in early and middle adolescence.

### Bullying and (Lack of) Defending of Victims in the Peer Group

Four decades of research on bullying has brought considerable insight into its prevalence, correlates, antecedents, and consequences among students (Hymel and Swearer, 2015). Bullying is stubbornly persistent and widespread in schools across the world (Chester et al., 2015) to the detriment of the victims who tend to suffer from social, emotional, and physical health problems (Rivara and Le Menestrel, 2016). Bullying is a complex social phenomenon and part of a social ecology involving all students in a social setting (Salmivalli, 2010). The participant roles approach considers the different roles that students have in the bullying process. Peer witnesses are present when bullying takes place and it matters how they respond. Some students choose to side with the bullies, and act as assistants or reinforcers (e.g., by joining in or making fun of victims), whereas other students behave in prosocial ways on behalf of the victims, and act as defenders of victims (e.g., those who help or comfort them), or remain bystanders (e.g., those who withdraw). Students who value social status might join the bullies as a way to enhance their own social status in the group (Rambaran et al., 2020), whereas students who empathize with the victims might choose to help them (Lambe et al., 2019).

The fact that many victims do not have a defender—even though most bystanders empathize with them and disapprove of bullying (Salmivalli, 2010)—suggests there are certain socio-cognitive processes at work that influence students’ defending or bystanding choices. Indeed, it has often been speculated that defending is demanding for many students as that puts them at risk of becoming victims themselves (Pozzoli et al., 2012). This is because defenders are challenging the status and power of the bullies by siding with the victim, and because of that bullies may retaliate against them (Salmivalli et al., 2011). Many students may therefore feel they are powerless against bullies, making it more difficult for them to intervene, and as such, it would be less risky to remain a passive bystander instead. A consequence of this is that in many cases defending of victims is unlikely or unsuccessful (Hawkins et al., 2001). This often results in the continuation of bullying.

### Socio-Cognitive Processes of Defending and Bystanding

One important social cognition that may influence students’ defending or bystanding choices is social competence beliefs—that is, students’ perceptions about their ability to behave effectively in social situations (Di Giunta et al., 2010). This can be understood by self-efficacy theory, which relates to the individual’s beliefs in their level of functioning (Bandura, 1997). In order to decide to do something people have to believe that they can perform the specific behavior to achieve their goal. In the context of bullying, students’ self-efficacy in defending may depend, in particular, on their beliefs in their capacity to act successfully in tackling bullying. What may differentiate defenders from bystanders is their self-efficacy for defending. Research has demonstrated that students with a higher degree of (either social or defending) self-efficacy defend more and that defenders have higher self-efficacy than bystanders (Lambe et al., 2019). One explanation is that bystanders generally do not know how to be successful in social situations involving conflict or how to defend the victims, more specifically, because they have never practiced it (Gini et al., 2008), whereas those who have had practice probably acquired self-confidence or self-esteem to stand up to the bullies (Pöyhönen et al., 2010). This suggests that not only can students reflect on their self-efficacy (Pronk et al., 2013), but that defending can shape students’ self-efficacy beliefs, too. Students may believe they can be effective in bullying situations because they already have resorted to direct (e.g., confronting the bully) or indirect intervention (e.g., consoling the victim or warning the teacher). However, we lack insight into how defending self-efficacy develops because this has rarely been the focus of investigation.

Another important social cognition to consider in the defending-bystanding process that has been more recently proposed in the bullying literature is perceived moral distress, a moral construct that can be defined as “painful feelings or psychological distress that occur when a person is conscious of the morally appropriate action but cannot carry out that action because of external or situational obstacles” (Forsberg et al., 2014, pp. 568–569). While limited research exists that examines the role of moral distress, it has been found that, like defending self-efficacy, also perceived moral distress is a positive correlate of defending of victims (Gini et al., 2020). In the extant literature, helping-bystanding behavior has typically been explained by bystander theory (Darley and Latané, 1968), which involves recognizing that someone is in distress, considering it to be an emergency, and a personal responsibility to intervene (Pozzoli and Gini, 2012). Most bystanders recognize that victims are distressed and need support (Salmivalli, 2010), and that defending is expected from them. However, even when bystanders feel that they should intervene and know what they should do, they may be constrained by a personal failing—for example, fear—or by situational factors, such as group norms (McCarthy and Deady, 2008). Bystanders who are distressed by this incapacity (e.g., “I feel bad because I should have done something to help but I didn’t”) may be motivated to defend in subsequent occasions to avoid the negative emotions that may be associated with inaction (Gini et al., 2020). Indeed, it has been shown that adolescents who anticipated guilt and shame for not helping a victim were more likely to defend (Pronk et al., 2017). However, like defending self-efficacy, we lack an understanding of how bystanding moral distress develops in individuals as this has rarely been the focus of interest.

### Friend Influence in Defender Self-Efficacy and Bystander Moral Distress

When students are in a school setting that offers opportunities for friendships with peers, it is important to consider to which extent friends can influence individuals’ social cognitions and emotions about defending. Adolescence is a critical period to examine this because adolescents attach great value to developing positive relationships with peers during this time (Poulin and Chan, 2010). Accordingly, adolescents are more attuned to, sensitive, and susceptible to the behaviors and attitudes of peers, in particular friends (Brechwald and Prinstein, 2011). Indeed, research has consistently demonstrated that adolescents’ values and beliefs are influenced by their peers, in particular that students are inclined to adopt the values and beliefs of their friends (Brechwald and Prinstein, 2011). Social cognitive theory (Bandura, 1986, 2001) provides a framework for understanding this process. According to this perspective, through social interactions with and observations of others, adolescents acquire skills, strategies, beliefs, behaviors, values, and emotions that shape themselves and others around them. Since bullying is a group phenomenon and occurs in the presence of other students rather than in isolation (Salmivalli, 2010), it is reasonable to assume that social cognitions and emotions of defending are affected by adolescent peers, in particular friends, too. For instance, friends may share their experiences concerning defending, such as instances where they successfully defended the victims, or instances where they failed to do so. Sharing this information among friends will convey to friends which defending strategies are fruitful. Thus, friends may learn from each other’s experiences, and based on their friends’ experiences may decide which defending strategies to adopt as they will be effective. Through peer influence friends may encourage defending in each other (Veenstra and Huitsing, 2021), for instance, by promoting positive values and beliefs about defending. Friends may also share with each other how they felt about their defending experiences, for instance, whether helping the victims brought them joy, or concerns for the victims when they could not. In this perspective, exposure to friends’ explicit expressions of concerns or emotions for undefended victims may shape one’s own concerns and emotions. Following this line of reasoning, it is to be expected that friends would influence each other’s social cognitions of defending and bystanding (i.e., perceptions of self-efficacy and moral distress).

## Current Study

An important research gap that still exists is a limited understanding of socio-cognitive and peer-group processes of defending and bystanding. To contribute addressing these issues, the aim of the current study was to examine the relation between socialization and (social cognitions of) defending and bystanding. Based on the tenets of social cognitive theory and on previous studies reviewed, it was expected friendships with peers to be important in the socialization of (social cognitions of) defending and bystanding. In particular, it was expected that not only do friends influence defending and bystanding in each other, but they also influence one another’s beliefs and emotions about their (in)abilities to defend the victims of bullying. If there is socialization on (social cognitions of) defending and bystanding in friend networks, then this would serve as the impetus for research that investigates how this comes about. Note that this study did not evaluate the process through which influence occurs. Rather, this study was aimed at testing whether the change in (social cognitions of) defending and bystanding exhibits a pattern consistent with socialization. To substantiate this claim, the ancillary analyses included an effect that captured the average behavior (i.e., either defending or bystanding) or average perception (i.e., either self-efficacy or moral distress) of all classmates and a comparison of the relative importance of this effect against the effect of friend influence. By clarifying the nature of the longitudinal association between friendship and (social cognitions of) defending and bystanding, it would make it possible to identify where future research and intervention efforts should be targeted (i.e., to understand how socialization occurs).

## Method

### Sample

Data for the current study were drawn from a larger project focused on the role of social-cognitive and moral processes in adolescent development. Part of that dataset has been previously used in two other studies that focused on defending behavior in bullying (Gini, Pozzoli et al., 2022) and aggression (Gini, Thornberg et al., 2022) as outcomes. There is some overlap between the data used in the two previous studies and those used in the current study: main variables (i.e., defending, bystanding, self-efficacy, or moral distress) were used in all three studies to some capacity, but the focus on socialization of (social cognitions of) defending and bystanding in the current study addresses a clearly distinct research question. Further note that previous studies examining some of these variables together found significant associations between them (e.g., self-efficacy and moral distress both enhance defending in individuals; see Gini et al., 2020), and were thus important to control for to obtain more robust estimates of social influence for each behavior and perception. Though these are discussed generally, they are not the aim of the current study.

Data were collected from nine public schools located in urban and suburban areas in the North of Italy at two consecutive waves (W) over a 6-month period. W1 took part in December 2017, after about 3 months since the beginning of the school year; W2 took part about 6 months later, at the end of May 2018 (almost at the end of the school year). In total, 67 seventh- to tenth-grade classrooms were involved in the study across both waves (in Italy, students typically enter seventh grade when they are 12 years old).

In the Italian school setting, the classroom represents the most meaningful unit of peer clustering and thus is considered a very stable social context for the development of youth and their relationships with peers at school. Students stay in the same classroom with the same peers every day for the whole school year, irrespective of the subject to be taught. Hence, students’ relationships with peers are typically observed within the same classroom rather than outside of the classroom. This, together with the small number of schools included in the sample, is the reason why friendships and (social cognitions of) defending and bystanding were measured at the classroom-level rather than at the school-level.

Of the 1562 students across 67 classrooms in 9 schools who were eligible to participate in the study, 208 students (13.3%) did not participate at both waves, and because there was no information on them, they were excluded. Of the remaining 1354 students (51.3% boys), 54 students (4.0%) did not participate at W1 and 122 students (9.0%) did not participate at W2. The average classroom size was 20 (minimum = 10; maximum = 30). The 67 classrooms consisted of 16 grade 7 classrooms (*n* = 312), 16 grade 8 classrooms (*n* = 292), 19 grade 9 classrooms (*n* = 420), and 16 grade 10 classrooms (*n* = 330). The number of classrooms within schools varied between 3 and 13. Students came primarily from middle-to-high class families (FAS III scale), and most students were Italian (based on the father’s country of origin: 81.39%; 9.08% were other European, 1.85% were Arabic, 1.40% were Asian, 1.11% were African, and 1.03% had other nationalities; the remaining 4.14% did not know/report the country of origin of their fathers for whatever reason).

### Procedure

Participation of classrooms in the study was first authorized by school principals. Then, participants’ parents provided an active consent for participation in both waves. Less than 10% of students in the participating classrooms did not receive parental consent. Before each data collection session, assent for participation was also obtained from each of the students with parental consent. They were informed that participation in the study was voluntary and that they could refuse to participate or withdraw from the study at any time. None of the participants refused to participate.

Data were collected twice in each classroom during one school year; the participants filled out a web-based questionnaire on computers in their regular classrooms. W1 and W2 data were matched with an anonymized alphanumeric code. A graduate research assistant was present during the data collection sessions and informed the participants that their answers would be treated anonymously and that they could raise their hand if they needed assistance (e.g., to clarify items or words of the questionnaire). At the end of each data collection session, any questions about the content of the questionnaires or the general aims of the study were answered. The study protocol was approved by the Ethics Committee for Research in Psychology at the University of Padua (protocol #1157/2012) and was conducted in line with the principles of the Declaration of Helsinki.

### Measures

#### Friendship networks

At each observation, participants were asked to nominate their friends in their classroom—“Who are your friends in your classroom?” Students could choose as many same-sex and other-sex classmates as they wished. Students were presented with a roster showing the names of all their classmates at the particular wave. Friendship networks consisting of directed friendship nominations were constructed for each classroom (1 = a given nomination, 0 = absent nomination). Missing values due to students leaving the schools over time were coded as 10 (structural missing), which enabled us to control for composition changes in the schools; missing values due to any other reason were coded as “NA” (regular missing). Changes in school composition were minimal (7 students left a school at W2).

#### Defending and bystanding

Defending and bystanding were measured with the Students’ Behavior during Bullying Episodes Scale (Pozzoli et al., 2016). A complete list of items for each measure can be found in Table S1 in the Online Supplements. In line with the participant roles approach (see e.g., Kärnä et al., 2013, p.540), a person would be considered defending the victim when he or she “comforts the victim or encourages him/her to tell the teacher about the bullying,” “tells the others to stop bullying,” and “tries to make the others stop bullying.” Defending thus consists of multiple components, and they all contribute to it. The defending measures used in the current study incorporate all these aspects to be able to relate to current understanding of peer defending in the bullying literature (e.g., Lambe et al., 2019). After providing a definition of bullying, four items were used to assess defending (e.g., “I defend classmates who are targeted by gossip or false rumors that are said behind their back”) and four items measured passive bystanding (e.g., “If I know that someone is being excluded or isolated from the group, I act as if nothing has happened”). Participants were asked to rate how often, during the last 3 months, they engaged in each of the described behavior on a 5-point scale (e.g., 1 = *Never*, 5 = *Almost always*). The scale has been extensively used with Italian adolescents and demonstrated good psychometric qualities (e.g., Pozzoli et al., 2017). For each participant, responses to the respective items for each behavior were averaged and rounded to obtain a score of defending and bystanding at each wave (Cronbach’s αs: 0.80 at both waves for defending; 0.62 at W1 and 0.68 at W2 for bystanding). Note that scores were rounded because the estimation method (RSiena) requires that dependent variables have whole values. Recently, the method has been extended to be used with continuous dependent behavior variables (Niezink et al., 2019). However, the effect parameter used to capture socialization of (social cognitions of) defending and bystanding in the current study (i.e., average similarity effect), corresponding to adoption of friends’ mean scores (how it was conceptualized in this study), is not yet implemented.

#### Defending self-efficacy

Self-efficacy was measured with a 9-item scale (adapted from Barchia and Bussey, 2011) that measure three forms of helping behavior (i.e., consoling the victim, telling the bully to stop, and encouraging the victim to report the bullying to an adult) for each type of bullying or victimization (i.e., physical, verbal, and relational). A sample item is the following: “How easy is it for you to console a classmate who has been hit, kicked or punched?” (See Table S1 for a complete list of items.) Participants were asked to rate their sense of self-efficacy for defending on a 6-point scale (e.g., 1 = *Not at all*, 6 = *Very much*). For each participant, responses to the items were averaged and rounded to obtain a score of defending self-efficacy at each wave (Cronbach’s αs: 0.91 at W1 and 0.92 at W2).

#### Bystanding moral distress

Moral distress was measured with a scale that has been recently developed for Italian adolescents (Gini et al., 2020). Participants were instructed to carefully read a scenario about bullying: “Sometimes in schools one or more students hurt or harass another student who cannot defend himself or herself. Examples of this are teasing, mocking, threatening, hitting, name-calling, or freezing out from the group in a way that makes the student sad, upset, or afraid. Try to remember situations in your school in which you have seen this happening and in which you, for some reason, did not help the student being targeted. How did you feel when you did not help the victim?” Afterwards, 7 items were presented (e.g., “I felt very bad because I should have helped the student but I couldn’t”, “I felt really distressed because I didn’t dare to help the victim”; see Table S1 for a complete list of items) and participants were asked to rate the extent to which each statement was true for them on a 5-point scale (e.g., 1 = *Completely untrue*, 5 = *Completely true*). This scale has been previously used with Italian adolescents and showed good psychometric qualities (Gini et al., 2020). For each participant, responses to the items were averaged and rounded to obtain a score of bystanding moral distress (Cronbach’s αs: 0.75 at W1 and 0.77 at W2).

#### Demographic and control variables

Sex was coded 1 = boys, 0 = girls.

### Analytic Strategy

This study applies a stochastic actor-oriented model (SAOM) implemented in the Simulation Investigation for Empirical Network Analysis software package (i.e., RSiena version 1.2-23; Ripley et al., 2021). Here, the focus is on the effects used to test the hypothesis. The interested reader is referred to the Online Supplements for a brief description of the SAOM and other findings. The model has a friend (network) selection component (function) and a separate behavior change and a separate perception change component (because defending and self-efficacy were analyzed together in the same model, and bystanding and moral distress were analyzed together in another model). The effects in the behavior or perception function used to predict change in behavior or perception are explained first. Friend influence is modeled using the Average Similarity effect (in RSiena terms). This effect tests whether students adjusted their behavior or perception to the mean of their friends. This effect captures both increases in behavior or perception in response to friends with higher levels of the behavior or perception, as well as decreases in behavior or perception in response to friends with lower levels of the behavior or perception than the adolescent. This measure assumes equal influence regardless of the number of friends that the adolescent has—on average, students had about 10 to 11 friends. Note that this is on the larger size (relative to classroom size, which was on average 20 students) than what would typically be expected for an adolescent sample. An unrestricted nomination procedure may have contributed to this. It is also possible that by providing a list of names to students it was easier for them to nominate their friends rather than to recall from memory. Prior to the SAOM analyses, it was assessed whether the friend networks adhered to what can be expected of adolescent friend networks in terms of key network features, such as reciprocity, transitivity and gender homophily (see Veenstra et al., 2013). It was found that the networks were not so different from other US and non-US samples, as they demonstrated high reciprocity and high transitivity and strong gender homophily (see Table 2). Nonetheless, to minimize the influence that the number of friends might have on change in behavior or perception, an effect of indegree (in RSiena terms) was included. Moreover, an effect of classroom size was included to account for the possibility that smaller classrooms tend to be more cohesive (contain relatively more friendship ties). Linear and quadratic shape parameters were used to capture the distribution of the behavior or perception over time; the models also accounted for students’ sex for more robust estimates of social influence on change of behavior or perception.

To examine friend selection on behavior or perception, model specification in the network function was aligned such it mirrored that of the behavior or perception function. Friend selection based on behavior or perception was expected to occur when youth (ego, representing the focal student) selected their friends (alters, representing the nominated friends) based on similarity in behavior or perception, captured with the similarity effect (in RSiena terms). For more reliable estimates of friend selection processes, the main effects were adjusted for as represented by a separate ego effect and a separate alter effect for behavior or perception. Additional controls were sex, classroom size (as mentioned above), and a set of network effects (e.g., reciprocity, transitivity, network activity or popularity; for a complete list of effects and their interpretation, Table S2) that are important predictors of friendship change, which improve fit and the robustness of friend selection on behavior or perception.

Because the models were too complex to be estimated with the small classrooms (resulted in unsatisfactory convergence) a pooled model was estimated that constrained estimates to be equal across classrooms in each school while recognizing that friendships were not possible between students in different classrooms within the same school (using structural zeroes, i.e., 10 for students in dyads from different classrooms). The parameter estimates were summarized with a meta-analysis using the Snijders and Baerveldt method (2003), which provides a model showing the mean estimates across all schools, including a test for heterogeneity between schools.

## Results

### Descriptives

Means of and correlations between the study variables are provided in Table 1. Sex differences indicate that, compared to boys at both waves, girls scored higher on defending (*t* = 7.7 at W1 and 7.4 at W2), self-efficacy (*t* = 5.0 at W1 and 5.4 at W2), and moral distress (*t* = 11.3 at W1 and 10.9 at W2), and lower on bystanding (*t* = ‒5.4 at W1 and ‒5.5 at W2; all *p*s < 0.01).

**Table 1:** Means, standard deviations of, and correlations between the study variables per wave

	Boys/Girls							
Variable	1	2	3	4	5	6	7	8
1. Defending W1	―	0.51**	0.41**	0.26**	‒0.25**	‒0.22**	0.20**	0.15**
2. Defending W2	0.49**	―	0.33**	0.38**	‒0.23**	‒0.35**	0.13**	0.20**
3. Self-efficacy W1	0.45**	0.26**	―	0.48**	‒0.20**	‒0.19**	0.08*	0.06
4. Self-efficacy W2	0.36**	0.39**	0.49**	―	‒0.16**	‒0.19**	0.06	0.14**
5. Bystanding W1	‒0.19**	‒0.26**	‒0.15**	‒0.14**	―	0.43**	‒0.19**	‒0.09*
6. Bystanding W2	‒0.24**	‒0.27**	‒0.18**	‒0.17**	0.36**	―	‒0.10**	‒0.14**
7. Moral distress W1	0.27**	0.26**	0.19**	0.07^†^	‒0.10**	‒0.10**	―	0.67**
8. Moral distress W2	0.25**	0.32**	0.21**	0.21**	‒0.08*	‒0.15**	0.56**	―
Mean girls	3.35	3.21	3.97	3.94	2.05	2.10	3.66	3.62
Mean boys	2.97	2.86	3.69	3.63	2.27	2.34	3.13	3.10
*SD* girls	0.87	0.84	0.98	0.99	0.74	0.74	0.84	0.80
*SD* boys	0.90	0.85	1.00	0.99	0.78	0.81	0.82	0.86
Diff. girls-boys^a^	7.7**	7.4**	5.0**	5.4**	‒5.4**	‒5.5**	11.3**	10.9**
ICC classroom	0.09	0.05	0.04	0.02	0.04	0.05	0.25	0.24
ICC school	0.05	0.02	0.01	0.02	0.01	0.01	0.22	0.23

Correlations for boys are reported below the diagonal; correlations for girls are reported above the diagonal W1 = Wave 1; W2 = Wave 2

*ICC* = intraclass correlation

^†^*p* ≤ 0.10; *p ≤ 0.05; **p ≤ 0.01

^a^Mean difference between boys and girls calculated using independent samples *t*-tests

For both boys and girls, scores on defending (*r* = 0.49 for boys and 0.51 for girls), self-efficacy (*r* = 0.49 for boys and 0.48 for girls), bystanding (*r* = 0.36 for boys and 0.43 for girls), and moral distress (*r* = 0.56 for boys and 0.67 for girls) between the two waves were moderate stable over time, as indicated by moderate positive correlations (all *p*s < 0.01). Defending correlated positively with self-efficacy and moral distress, and negatively with bystanding, whereas self-efficacy correlated positively with moral distress and negatively with bystanding; finally, bystanding correlated negatively with moral distress. These patterns were similar for boys and girls (see Table 1).

A description of the averaged friendship networks and longitudinal transitions between the two waves of data are presented in Table 2. About one-quarter of friendship nominations had the same defending scores and self-efficacy, whereas two-fifth of nominations had the same bystanding and moral distress, indicating that friends showed modest similarity in these behaviors and perceptions. In line with social network analysis requirements (Veenstra et al., 2013), a sufficient amount of change was observed with regard to friendship nominations (i.e., Jaccard index was around 61%) and each of the behaviors and perceptions (i.e., fraction stable actors varied between 41% and 53%).

**Table 2:** Description of the sample and the variables per measurement and longitudinal transitions between measurements

Variable		W1	W2	Change	W1‒W2
Sample				Sample change	
Cohort size		1300	1226	Number of leavers	7
Respondents missing		54	128	Number of joiners	0
Fraction boys		52%	51%	Number of stayers	1347
Friendship				Friendship change	
Average outdegree		10	11	Hamming distance	686
*SD* outdegree		6	6	Jaccard index	0.61
*SD* indegree		4	4	Defending change	
Density		8%	8%	Distance	86
Reciprocity		68%	69%	Fraction stable	45%
Transitivity		71%	76%	Self-efficacy change	
Same sex		64%	63%	Distance	95
Same defending		29%	28%	Fraction stable	41%
Same self-efficacy		27%	27%	Bystanding change	
Same bystanding		38%	39%	Distance	79
Same moral distress		40%	38%	Fraction stable	49%
				Moral distress change	
				Distance	66
				Fraction stable	53%

W1 = Wave 1; W2 = Wave 2. Density is the proportion of given ties relative to the total amount of possible ties; Reciprocity is the proportion of mutual ties; Transitivity is the proportion of tie configurations that could become cohesive peer groups; Hamming distance is the amount of tie changes from the beginning to the end of the measurements (i.e., between the two waves); Jaccard index is the fraction of stable ties relative to all new, lost, and stable ties

### SAOM Analysis

To examine the relation between socialization and (social cognitions of) defending and bystanding, the focus is on the results in the behavior and perception change functions of the SAOM that relate to friend influence on the behaviors and perceptions (Model 1 in Table 3 contains results of defending and self-efficacy; Model 1 in Table 4 contains results of bystanding and moral distress).

**Table 3:** Directed network models predicting selection and influence on defending and self-efficacy

	Model 1: Main model				Model 2: Classroom norm model			
Parameter (statistic)	Est.		(SE)	*n*	Est.		(SE)	*n*
Friendship function								
Rate of network change	10.76	***	(1.67)^a^	9	10.76	***	(1.67)^a^	9
Outdegree	‒2.06	***	(0.15)	9	‒2.07	***	(0.16)	9
Reciprocity	1.97	***	(0.39)^a^	9	1.97	***	(0.38)^a^	9
Transitive triplets	0.28	***	(0.06)^a^	9	0.28	***	(0.06)^a^	9
Reciprocated transitive triplets	‒0.29	**	(0.07)^a^	9	‒0.29	**	(0.07)^a^	9
Indegree popularity	‒0.04		(0.03)^a^	9	‒0.04		(0.03)^a^	9
Outdegree activity	0.02		(0.01)^a^	9	0.02		(0.02)^a^	9
Same sex	0.35	***	(0.05)	9	0.35	***	(0.05)	9
Classroom size	‒0.89	^†^	(0.47)^a^	9	‒0.92	^†^	(0.48)^a^	9
Defending alter	‒0.02		(0.02)	9	‒0.02		(0.02)	9
Defending ego	‒0.01		(0.06)^a^	9	‒0.01		(0.06)^a^	9
Defending similarity	0.41		(0.31)	9	0.40		(0.30)	9
Self-efficacy alter	0.01		(0.02)	9	0.01		(0.02)	9
Self-efficacy ego	‒0.02		(0.03)	9	‒0.02		(0.03)	9
Self-efficacy similarity	0.08		(0.19)	9	0.07		(0.19)	9
Defending function								
Defending: rate of change	1.92	***	(0.11)	9	1.93	***	(0.12)	9
Defending: linear shape	‒0.12		(0.17)	9	‒0.13		(0.19)	9
Defending: quadratic shape	‒0.35	***	(0.05)	9	‒0.33	***	(0.07)	9
Defending: average similarity	1.64	*	(0.50)	8	2.09	*	(0.78)	8
Defending: indegree	0.00		(0.02)	9	0.00		(0.02)	9
Defending: sex (ref. = girl)	‒0.12		(0.08)	9	‒0.12		(0.09)	9
Defending: self-efficacy	0.17	**	(0.05)	9	0.17	*	(0.05)	9
Defending: bystanding	‒0.23	**	(0.06)	9	‒0.23	*	(0.07)	9
Defending: moral distress	0.22	*	(0.08)	9	0.20	**	(0.06)	9
Defending: classroom average^b^					‒0.13		(0.18)	9
Self-efficacy function								
Self-efficacy: rate of change	2.21	***	(0.22)	9	2.20	***	(0.21)	9
Self-efficacy: linear shape	‒0.13		(0.10)	9	‒0.14		(0.11)	9
Self-efficacy: quadratic shape	‒0.25	***	(0.05)	9	‒0.26	**	(0.07)	9
Self-efficacy: average similarity	1.76	^†^	(0.81)	9	1.46		(1.12)	9
Self-efficacy: indegree	0.01		(0.01)	9	0.01		(0.01)	9
Self-efficacy: sex (ref. = girl)	‒0.14	^†^	(0.07)	9	‒0.13	^†^	(0.06)	9
Self-efficacy: defending	0.20	*	(0.06)	9	0.20	*	(0.07)	9
Self-efficacy: bystanding	‒0.07		(0.07)	9	‒0.09		(0.07)	9
Self-efficacy: moral distress	0.01		(0.05)	9	0.00		(0.05)	9
Self-efficacy: classroom average^b^					‒0.07		(0.21)	9

Following recommendations provided by the RSiena manual (Ripley et al., 2021), a threshold of 4 or 5 for the standard error was met for most parameters; for the “average similarity” effect for behavior (perception) dynamics, estimates and standard errors tend to be larger, and a larger threshold (10) was appropriate. In some cases, the standard error for a parameter exceeded this threshold and was left out of the meta-analysis. Setting a higher threshold (i.e., including omitted cases) yielded substantially the same results as those presented here (available upon request)

^†^*p* < 0.10; *p < 0.05; **p < 0.01; ***p < 0.001 (two-tailed)

^a^Significant differences between schools according to the Snijders and Baerveldt (2003) method

^b^This was captured by the average behavior or perception of all students in a classroom (variable was mean centered around the school’s average), reflecting a classroom descriptive norm (see e.g., Rambaran et al., 2021)

**Table 4:** Directed network models predicting selection and influence on bystanding and moral distress

	Model 1: Main model				Model 2: Classroom norm model			
Parameter (statistic)	Est.		(SE)	*n*	Est.		(SE)	*n*
Friendship Function								
Rate of network change	10.65	***	(1.53)^a^	9	10.64	***	(1.53)^a^	9
Outdegree	‒2.02	***	(0.16)	9	‒2.02	***	(0.16)	9
Reciprocity	2.02	***	(0.38)^a^	9	2.01	***	(0.37)^a^	9
Transitive triplets	0.28	**	(0.06)^a^	9	0.28	**	(0.06)^a^	9
Reciprocated transitive triplets	‒0.29	**	(0.07)^a^	9	‒0.29	**	(0.07)^a^	9
Indegree popularity	‒0.05		(0.03)^a^	9	‒0.04		(0.03)^a^	9
Outdegree activity	0.02		(0.01)^a^	9	0.02		(0.02)^a^	9
Same sex	0.37	***	(0.05)^a^	9	0.37	***	(0.05)^a^	9
Classroom size	‒0.93	^†^	(0.48)^a^	9	‒0.90	^†^	(0.46)^a^	9
Bystanding alter	0.14	^†^	(0.06)^a^	9	0.13	^†^	(0.06)^a^	9
Bystanding ego	‒0.02		(0.07)^a^	9	‒0.02		(0.07)^a^	9
Bystanding similarity	0.17		(0.28)	9	0.13		(0.29)	9
Moral distress alter	‒0.02		(0.06)	9	‒0.02		(0.06)	9
Moral distress ego	0.00		(0.03)	9	0.00		(0.03)	9
Moral distress similarity	‒0.39	*	(0.13)	9	‒0.36	*	(0.12)	9
Bystanding Function								
Bystanding: rate of change	2.00	***	(0.21)	9	1.98	***	(0.21)	9
Bystanding: linear shape	‒0.09		(0.19)	9	‒0.10		(0.20)	9
Bystanding: quadratic shape	‒0.26	**	(0.07)	9	‒0.28	**	(0.06)	9
Bystanding: average similarity	2.34	**	(0.69)	9	2.26	*	(0.70)	9
Bystanding: indegree	0.01		(0.02)	9	0.01		(0.02)	9
Bystanding: sex (ref. = girl)	0.10		(0.10)	9	0.09		(0.10)	9
Bystanding: moral distress	‒0.05		(0.11)	9	‒0.05		(0.11)	9
Bystanding: defending	‒0.20	*	(0.07)	9	‒0.19	*	(0.08)	9
Bystanding: self-efficacy	‒0.04		(0.05)	9	‒0.04		(0.05)	9
Bystanding: classroom average^b^					‒0.07		(0.22)	9
Moral Distress Function								
Moral distress: rate of change	1.69	***	(0.11)	9	1.69	***	(0.10)	9
Moral distress: linear shape	‒0.01		(0.19)	9	‒0.01		(0.19)	9
Moral distress: quadratic shape	‒0.30	***	(0.06)	9	‒0.24	*	(0.08)	9
Moral distress: average similarity	3.80	*	(1.50)	7	4.69	*	(1.62)	7
Moral distress: indegree	0.01		(0.02)	9	0.01		(0.02)	9
Moral distress: sex (ref. = girl)	‒0.43	*	(0.17)	9	‒0.45	*	(0.18)	9
Moral distress: bystanding	‒0.17		(0.15)	9	‒0.06		(0.22)	9
Moral distress: defending	0.15	^†^	(0.08)	9	0.16	^†^	(0.08)	9
Moral distress: self-efficacy	0.10	*	(0.04)	9	0.11	*	(0.04)	9
Moral distress: classroom average^b^					‒0.43	*	(0.17)	8

See notes Table 3.

#### Influence dynamics

Students’ behaviors and perceptions changed in relatively similar ways across schools, as effects did not vary between schools. The negative *quadratic shape* effects indicate that change in behaviors and perceptions followed a curvilinear trend: adolescents with higher initial values decreased and those with lower initial values increased over time (a self-correcting effect, or regression to the mean). Change in behaviors and perceptions depended on students’ attributes. Note that the contribution of each attribute is net of all other effects in the models. As expected (given previous research), higher self-efficacy and moral distress resulted in higher defending over time, whereas bystanding resulted in lower defending. Higher defending resulted in higher self-efficacy and boys developed lower self-efficacy than girls. Further, higher defending resulted in lower bystanding. While higher defending and self-efficacy both resulted in higher moral distress generally, boys developed lower moral distress than girls.

Taking everything else into account, in line with the hypothesis change in behaviors and perceptions was explained by friend influence: The estimate of the *Average Similarity* effect capturing influence from friends was positive and significant for defending (*b* = 1.64, *p* < 0.05) and self-efficacy (*b* = 1.76, *p* = 0.06) as well for bystanding (*b* = 2.34, *p* < 0.01) and moral distress (*b* = 3.80, *p* < 0.05). This means that adolescents were more likely to adopt their friends’ behaviors and perceptions over time. To gain insight into their importance, the procedure outlined in Indlekofer and Brandes (2013) is useful which calculates the relative importance (RI) of the effect of friend influence against the other effects included in the behavior or perception function of the models. Because RI scores will change based on model specification, this exercise is worthwhile only to the extent that models are well-specified (Schaefer et al., 2022). The results are shown in Table  S3, which reveals that RI scores were rather small for defending (9.5%) and self-efficacy (11.7%), and though twice as large for bystanding (18.8%) and moral distress (22.5%), effects of individual covariates (26.5‒28%) and in particular those representing general tendencies (50.9‒63.7%) were noticeably larger. This indicates that the effect of friend influence to behavior or perception change was small overall but still significant.

#### Selection dynamics

There was no effect of similarity for defending, self-efficacy, and bystanding (non-significant *similarity* effects), indicating that friend selection did not depend on similar levels of defending, self-efficacy, or bystanding (see Model 1 in Tables 3 and 4). A negative similarity effect was observed for moral distress (*b* = ‒0.39, *p* < 0.05). This indicates that adolescents befriended classmates with dissimilar values, and discontinued friendships with those having similar values. Thus, there is no evidence that students selected friends based on similar behaviors or perceptions. The non-significant main selection (*ego* and *alter*) effects further indicated that behaviors or perceptions were unrelated to sending and receiving friend nominations.

#### Ancillary analyses

The analyses lend support to the hypothesis that friends influence both behaviors and perceptions. However, given the high number of friends in relation to the average classroom size, the results might be reflective of classroom dynamics rather than socialization within friend networks. That is, it is possible that part of this effect can be explained by exposure and witnessing examples of successful or unsuccessful defending. At school, students are more likely to be exposed to the same events as their friends and less likely to be exposed to the same events as their non-friends (because friends hang out together). It is very possible that students become more similar to their friends in these behaviors and perceptions not because of socialization or influence processes, but because they are all exposed to the same things. To rule out the possibility that friends influence behaviors and perceptions rather than the average behaviors or perceptions of all classmates, it was tested to what extent the average of behaviors or perceptions in classroom explains change in individuals’ behaviors or perceptions. The intraclass correlation coefficients (see Table 1) indicated that a small portion of variation in defending (5–9%), self-efficacy (2–4%), and bystanding (4–5%), and a large portion of variation in moral distress (24–25%) was due to differences between classrooms, justifying further inspection. The results are presented in Model 2 in Tables 3 and 4. Estimates of friend influence were largely unaffected by the inclusion of the average of behavior or perception in classroom, except for estimates of friend influence for self-efficacy which was no longer significant due to increased uncertainty. Nonetheless, the relative influence remained largely the same (see Online Supplements, Table S3).

Though differences in ages from the youngest grade to the oldest are at least 4 years, it was not possible to account for grade due to limited variation at the school level. Schools had at most two grades, and schools were rather small generally, thus containing few classrooms. Importantly, schools had different grades, which made it impossible to compare the effect of grade in a meaningful way. To check the robustness of the results to assuming no grade differences, additional models were estimated that pooled information from all schools into one large network enabling an examination of grade (as exogenous predictor of change in behavior or perception; in these analyses, grade was coded such that it retained its numerical value: 7, 8, 9, and 10), while restricting friendships to the classroom with the additional assumption that friendship processes are similar between schools. These results offer substantively similar conclusions (see Online Supplements, Table S4).

As is common in peer relations research using stochastic actor-oriented modeling, the analyses were done with directed networks, which included friendships that were both reciprocal and non-reciprocal but, in these analyses, it did not matter for influence to occur whether those friendships were reciprocal or not. However, theory suggests that friendship reciprocity reflects cohesion and affinity between an adolescent target and influencer, thus making influence more likely to occur (Brechwald and Prinstein, 2011). Reciprocal friendships involve strong relationship qualities that enhance socialization effects. However, it is equally plausible that a nonreciprocated friendship with a desired peer makes adolescents more inclined to conform to this peer’s behavior (Heilbron and Prinstein, 2008). On average, two-thirds (68–69%; see Table 2) of the friend nominations were mutual, indicating that in most of the cases, both students in a friend dyad confirmed that a friendship between them was present. Still, one-third of friend nominations were on average directed to classmates that did not reciprocate the nomination, and it is possible that for these friendships the influence processes look different. To find out, friend influence was restricted to reciprocal friendships. This analysis included the reciprocated version of the Average Similarity effect (in RSiena terms), as well as the reciprocated version of the degree effect to control for the number of reciprocated friends. The results are presented in the Online Supplements, Table S5. There was no indication that friend influence processes operate differently in directed and undirected (reciprocated) friendships as the patterns were similar in both analyses.

## Discussion

By acknowledging that bullying is a group phenomenon and occurs in the presence of peer witnesses, developmental and peer relationships research have put great effort into understanding the role of peers in the bullying process in recent years (Salmivalli, 2010). However, most work has examined defending and bystanding and its antecedents without consideration of peer-network influences, with very few exceptions (Veenstra and Huitsing, 2021). Thus, researchers still know very little about the interpersonal mechanisms and peer group dynamics through which peers shape defending and bystanding behaviors. To advance the research in this area, this study investigated the extent to which friends can influence students’ defending and bystanding behaviors as well as their personal beliefs and emotions concerning these two behaviors. Findings provided support for this hypothesis, as friends’ defending and bystanding levels as well as their levels of self-efficacy and moral distress became more similar or were more likely to remain similar to each other.

These findings add to the growing body of research finding that peers, in particular friends, contribute to students’ defending and bystanding behaviors. Whereas previous research showed that peers generally affect defending and bystanding behaviors, for example, due to perceived peer normative pressure (Pozzoli and Gini, 2010) or peer group norms (Pozzoli et al., 2012), consistent with prior network research on defending behavior (Veenstra and Huitsing, 2021) the findings suggest that friends influence one another’s defending. Finding an effect of friend influence on bystanding behavior is novel, however, but aligns with network research finding friends also influencing the other bullying roles (i.e., bullying behavior and peer victimization; see Veenstra and Huitsing, 2021). Together, these studies provide evidence that bullying is a group process.

Research suggests that victims are especially in a vulnerable position in adolescence when bullying peaks (Kretschmer et al., 2017) and defending tends to diminish (Ma et al., 2019). Thus, if peer influence processes happen simultaneously for defending and bystanding roles, then this would provide mixed signals to students as some peers or friends promote defending behavior whereas others promote passive bystanding instead. Based on the findings, if an adolescent’s friends’ defending decreases (or their bystanding increases) over time, it would be expected that the adolescent’s defending decreases (or their bystanding increases) in tandem. Thus, friends can be both a protective and risk factor depending on whether they display high or low levels of defending and bystanding. Future research should clarify if adolescents are pulled more toward one set of friends than the other.

Whereas previous research focused on the social cognitions and emotions (e.g., self-efficacy, empathy, or morality) as individual characteristics of students that influence their defending and bystanding choices (Meter and Card, 2015), findings show that not only do friends influence one another’s defending and bystanding behaviors but also each other’s social cognitions and emotions concerning these behaviors, in particular their defending self-efficacy and bystanding moral distress. Although the relative effect size remained the same, the effect of friend influence for defending self-efficacy should be treated with caution as it was no longer significant when including the average of all classmates. Future research should clarify if friends are influential rather than all peers. Nonetheless, provisional findings suggest there is a more complex socialization process within friend (peer) networks. This process may involve friends (peers) exchanging information, and adopting and reinforcing one another’s beliefs and emotions about their (in)abilities to defend the victims of bullying in their classroom.

It is worth noting that defending consists of multiple components (i.e., comforting the victims, informing the teachers, and stopping the bullies) that are integral parts of defending (Kärnä et al., 2013). Like bullying, these vary in visibility and intensity and it is possible that the findings concerning friend influence effects differ by whether the bullying is violent (e.g., starting physical fights) or non-violent (e.g., spreading gossip, false rumors). Thus far, only one study looked at peer socialization of aggressive vs. nonaggressive defending in friend networks (Lambe and Craig, 2022), but it did not distinguish between aggressive vs. nonaggressive bullying. That study found that three out of four defending types examined, that is, defending by comforting, defending by reporting, and solution-focused defending were all socialized within friend networks, but socialization of aggressive defending was only present for a small group of students that engaged in this type of defending behavior. Research that looked at this for the variables that were investigated in the current study is currently unavailable and this would be an important avenue for future research to better understand socialization processes.

As with other studies (Meter and Card, 2015), the current study finds that both self-efficacy and moral distress promote defending behavior. Taking further into account that these are socialized within friend networks, then this indicates that adolescents are supported into defending. When there are friends from whom adolescents can learn and grow, and share their concerns, they may face these challenges together as friends experience the same things. However, most defending occurs among friends rather than non-friends or dislikes (Rambaran et al., 2021), and this explains why some victims remain undefended as they may not have a defender in their network.

Research suggests there are mental health implications to observing bullying at school: students who witness bullying are at increased risk of internalizing (e.g., anxiety and depression) and externalizing (e.g., substance use and hostility) problems (Rivers et al., 2009). The current study finds that defenders have both increased self-efficacy as well as increased moral distress. Thus, while defenders report to be more efficacious in defending the victims, they are also emotionally vulnerable when they decide, for any reason, to not defend them. One explanation is that defenders feel personally responsible for standing up to bullies and for the well-being of victims, and when some of the victims are left undefended because they cannot defend them all (and their focus is on defending friends rather than non-friends or dislikes), they experience moral distress while having a high degree of self-efficacy (Gini et al., 2020).

Finally, the current analyses revealed important sex differences: compared to boys, girls generally developed higher defending self-efficacy as well as higher bystanding moral distress. This aligns with previous studies showing that girls are generally higher in empathy than boys (Lambe et al., 2019), and in their perception more prepared and efficacious to intervene in bullying (Pronk et al., 2013). Future research should examine if boys and girls are susceptible to their friends’ self-efficacy and moral distress in distinct ways.

### Implications

The results from this study may have implications for anti-bullying intervention and programming in schools that aim to enhance defending in individuals (Salmivalli, 2010). First, the results can be used to better inform prevention and intervention efforts with adolescents. Not only do friends influence students’ defending and bystanding behaviors, this study shows that friends can also influence certain social cognitions and emotions associated with them, in particular self-efficacy in defending and moral distress in bystanding. Based on these findings, school administrators interested in enhancing defending (and diminishing bystanding) among their students should consider the role of peers, in particular friends, in shaping defending and bystanding behaviors as well as socio-cognitive processes associated with them. The findings suggest that students can learn from their peers (friends) in school or classroom how to be efficacious in defending situations, and at the same time can trigger emotional responses by each other when they are unable to defend the victims. Both of these socio-cognitive processes promote defending in individuals. Anti-bullying intervention and prevention researchers advocate that raising awareness and empathic understanding of the victim’s plight is crucial to reduce bullying, and self-efficacy for defending and moral distress are important factors contributing to defending (Salmivalli, 2010). Yet, the behaviors and perceptions of defenders and their relationships with victims do not occur in isolation, but exist in peer networks and in interplay with other relationships. In line with theories involving a “growth mindset” (Derr and Morrow, 2020) in the context of peers (i.e., a “peer mindset”; Sheffler and Cheung, 2019), peers may learn and grow from each other in their judgments of victims’ distress and how to be successful in defending. It is therefore important to incorporate how peer networks shape the behaviors, cognitions, and emotions of defenders and bystanders.

### Limitations and Directions for Future Research

The extent to which the findings are informative to anti-bullying interventions, however, is contingent on some caveats. First, how and why friend influence operates was not examined, and this may be necessary to develop more effective interventions and programming. Next, homogeneity in friend influence processes were assumed equal across classrooms within the same school, although differences in classroom levels of bullying and defending are common in early and middle adolescence (Salmivalli, 2010). Hence, analysis at the classroom level might have been preferable. Unfortunately, this was not viable in the current study due to failed model convergence for several classrooms, probably due to the small size of the classrooms and consequently low power, which is a known problem for these models (see e.g., Sijtsema et al., 2014). Though common practice, this study relied on self-reports which introduces the risk of socially desirable responses, especially for prosocial behavior such as defending behavior; hence, the use of peer reports would have been better for this. Moreover, the analyses were limited to two observations, and although students’ defending and bystanding behaviors as well as their perceptions of defending self-efficacy and bystanding moral distress changed within a short period of 6 months, this puts a constraint on making causal inferences about their development. Repeated measures are needed to more fully understand how these develop in the context of peers. Finally, and building on this, only two types of social cognitions were examined and there are other socio-cognitive factors (e.g., anti-bullying attitudes or empathy for victims) that may influence students’ defending or bystanding choices in peer networks.

## Conclusion

Past research suggests that peers are central to the understanding of defending and bystanding behaviors as the process by which this unfolds occurs in the presence of other students rather than in isolation. However, despite a dearth of studies on bystanders’ reactions to bullying in the peer group, limited research focusses on the social cognitions and peer-group processes that contribute to defending and bystanding behavior. The current study used longitudinal social network analysis to examine whether the development of (social cognitions of) defending and bystanding operates through socialization processes in friend networks. As expected, this study found evidence that friends can influence defending and bystanding behaviors as well as two social cognitions—self-efficacy and moral distress—associated with these behaviors in adolescence. Both self-efficacy and moral distress enhance defending, and these are all socialized within friend networks. However, findings concerning friend influence on self-efficacy are conditional as the average of classmates explained some of this effect, with no clear indication that students select friends based on similar levels of defending, bystanding, or any of the two social cognitions. Yet, novel findings highlight that rather than solely reflecting individual beliefs and emotions, beliefs and emotions concerning defending and bystanding are reinforced and shaped by peer-network influences.

## Supplementary Information

Supplemental Information
