# Supplementary material for: Socio-Cognitive Processes and Peer-Network Influences in Defending and Bystanding
Source: J Youth Adolesc. 2022 Jul 8;51(11):2077–91. doi: 10.1007/s10964-022-01643-z (PMC9508190; doi:10.1007/s10964-022-01643-z)
Supplement: Supplementary file 1 — Supplemental Information [file 10964_2022_1643_MOESM1_ESM.docx]

**Supplemental Material**

Socio-Cognitive Processes and Peer-Network Influences in Defending and Bystanding

Authored by:

J. Ashwin Rambaran, Tiziana Pozzoli, Gianluca Gini

**This file includes:**

Supplementary Text

Description of Network Properties and Individual Attributes

Brief Description of the SOAMs

Goodness of Fit

General Findings from SAOMs

Table S1 to S5

List of Items for the Behavior and Perception Measures

Parameters Used in the SAOMs

Results of Relative Importance Scores

Results of SAOMs with Grade

Results of SAOMs with Reciprocal Friends

References

**Supplementary Information Text**

**Description of Network Properties and Individual Attributes**

Table 2 presents the summarized network statistics for the nine schools. On average, adolescents nominated 10 to 11 friends across the schools (average degree). The majority of students nominated at least one friend (98–99%). The networks were characterized by high reciprocity and transitivity (see Table 2). About two-thirds (68–69%) of friendship nominations were reciprocated. Transitivity indexes the ratio of the number of actual and potential transitive triplets (71‒76%), reflecting the tendency to nominate friends of friends as friends. Almost two-thirds (63–64%) of friendships were same-sex.

**Brief Description of the SOAM**

In the SAOM, there are three functions that are estimated simultaneously, one predicting changes in dyadic friendship (friendship selection function), and two other predicting changes in behavior or perception (behavior or perception change function). In the friendship selection function, behavior (either defending or bystanding) or perception (either self-efficacy or moral distress) at the first wave is used to predict changes in dyadic friendship at the next wave. This provides information on the social selection of behavior or perception. Conversely, in the behavior or perception change function dyadic friendship at the first wave is used to predict changes in the dependent behavior (i.e., defending or bystanding) or perception (i.e., self-efficacy or moral distress) variable at the next wave. This provides information on the social transmission of behavior or perception. The SAOM thus allows researchers to examine processes of social selection and social influence simultaneously while controlling for each other, to allow for unbiased estimates of each process (Steglich et al., 2010).

**Goodness of Fit**

Convergence statistics for final models adhered to the standard criteria for convergence: all parameters had t ratios < 0.10; overall maximum convergence ratio < 0.25 (Ripley et al., 2021). The goodness of fit was acceptable. This was assessed with auxiliary statistics. Four auxiliary behavior and perception statistics were computed, and three auxiliary network statistics for the estimated models: defending distribution, self-efficacy distribution, bystanding distribution, moral distress distribution, outdegree distribution, indegree distribution, and triad census, by investigating how well these statistics are captured in a sample of networks simulated according to the estimated models (reported in Model 1 in Table 3 and Table 4). For each of these statistics, the differences between the values in the observed network (summed across the two waves of data for each school) and the estimated values for each model (summed across the 10,000 simulated networks) are assessed with the Mahalonobis distance (Ripley et al., 2021). Fit for a particular statistic is good or acceptable when the Mahalonobis distance is small. Assessment of goodness of fit with auxiliary statistics is essential to interpret the effects of main interest. Fit was satisfactory for out/indegrees, defending, self-efficacy, bystanding, and moral distress as the simulated values generally follow from the observed value of the statistics, though not optimal for certain values. Fit for triad census—representing local subgroup structures (friendship groups)—was generally speaking acceptable, but less so for the indirect connections inside these subgroups. In particular, shared outgoing and incoming ties were captured less well by the model parameters as well as some other more complex configurations. For an explanation of the different statistics of triad census, see Faust (2007).

**General Findings from SAOMs**

In this section, we describe the results of the full SAOMs reported in Table 3 and Table 4 that were not reported in the main text. These effects serve as controls for social selection and social influence on behavior or perception.

**Network Structure Effects**. The network effects were comparable across the two models (i.e., containing both defending and self-efficacy, and containing both bystanding and moral distress), though varied significantly between schools. The *network rate* effects indicate that the average number of changes in friendship nominations was between 10 and 11 between the two observations. In accordance with the low density of the school friendship networks, the negative *outdegree* (comparable with the intercept or grand mean in linear models) showed that adolescents were likely to be selective in their friendship nominations: They did not become friends with everyone. Friendship nominations were more likely when reciprocated, or part of a transitive triplet (positive *reciprocity* and *transitive triplets*). The latter suggests that adolescents were likely to nominate friends of their friends also as friends (but not reciprocally: negative *reciprocated transitive triplets*). Adolescents who initially received many nominations did not attract additional nominations over time (non-significant *indegree popularity*), nor did those who initially send many nominations become more active over time (non-significant *outdegree activity*), but there was significant variation in both between schools. Friendships were more likely between same-sex peers (positive *same sex*). Taken together, network structure gender homophily explained the friendship networks, though important variation exists between them.

| **Table S1**  List of Items for Each Behavior and Perception Measure. | | |
| --- | --- | --- |
| Measure | Classification^a^ | Item |
| Defending |  |  |
|  | Non-aggressive | 1. I help or comfort classmates who are excluded from the group and isolated |
|  | Non-aggressive | 1. I defend classmates who are targeted by gossip or false rumors that are said behind their back |
|  | Aggressive | 1. I defend the classmates who are hit or attacked hard |
|  | Aggressive | 1. I defend classmates who are threatened or offended |
| Self-efficacy |  |  |
|  | Non-aggressive | 1. How easy is it for you to console a classmate who has been hit, kicked or punched? |
|  | Non-aggressive | 1. How easy it is for you to console a classmate who is isolated, targeted by gossip or said bad things behind their back? |
|  | Non-aggressive | 1. How easy is it for you to console a classmate who receives bad nicknames, is threatened or to whom bad things are said behind their back? |
|  | Aggressive | 1. How easy is it for you to say to stop a classmate who beats, punches or pushes another classmate? |
|  | Aggressive | 1. How easy is it for you to stop a classmate who isolates others, gossip or says bad things about others behind their back? |
|  | Aggressive | 1. How easy is it for you to say to stop a classmate who gives bad nicknames to others, threaten others, or says bad things about others behind their back? |
|  | Non-aggressive | 1. How easy is it for you to encourage a classmate to tell a teacher or adult that they have been beaten or punched? |
|  | Non-aggressive | 1. How easy is it for you to encourage a classmate to tell a teacher or adult that they have been isolated, made fun of, or received bad comments? |
|  | Non-aggressive | 1. How easy is it for you to encourage a classmate to tell a teacher or adult that they have been receiving bad nicknames, threatened, or said bad things about? |
| Bystanding |  |  |
|  | Aggressive | 1. When a classmate is hit or pushed, I stand by and I mind my own business |
|  | Aggressive | 1. If a classmate is teased or threatened I do nothing and I don’t meddle |
|  | Non-aggressive | 1. If I know someone is excluded or isolated from the group I act as if nothing happened |
|  | Non-aggressive | 1. When a classmate is targeted by gossip or false rumors that are said behind their back, I do nothing and I mind my own business |
| Moral distress |  |  |
|  | — | 1. I felt very bad because I should have helped the student but I couldn’t |
|  | — | 2. I didn’t care because it wasn’t my business (reversed) |
|  | — | 3. I felt really distressed because I didn’t dare to help the victim |
|  | — | 4. I didn’t feel anything because it wasn’t my problem (reversed) |
|  | — | 1. I thought that the right thing to do was to help the student, but I wasn’t able to do that so I really felt bad |
|  | — | 1. I didn’t feel anything special (reversed) |
|  | — | 1. I felt guilty because I didn’t do anything to help the victim |
| Note. ^a^This distinguishes between aggressive vs. nonaggressive bullying for which defending, self-efficacy, or bystanding occurs (no distinction is being made for moral distress). | | |

| **Table S2**  Parameters in Directed Network Models Predicting Selection and Influence on Defending (Self-Efficacy) and Bystanding (Moral Distress). | | | |
| --- | --- | --- | --- |
|  | Configuration | | Interpretation {RSiena shortname} |
| Parameter (statistic) | Time *t* | Time *t* + 1 |  |
| **Friendship Function** |  |  |  |
| Rate of Change |  |  | Average number of opportunities of actors to change friendship ties (Rate) |
| Outdegree |  |  | Actor *i* extending friendship ties to alter *j* {density} |
| Reciprocity |  |  | Actor *i* reciprocating friendship ties to alter *j* {recip} |
| Transitive Triplets |  |  | Actor *i* extending friendship ties to alter *j* to whom they are indirectly tied (via *h*) {transTrip} |
| Reciprocated Transitive Triplets |  |  | Alter *j* reciprocating friendship ties to actor *i* to whom they are directly and indirectly tied (via *h*) {transRecTrip} |
| Indegree popularity |  |  | Actors with many incoming friendship ties attract more incoming ties {inPop} |
| Outdegree activity |  |  | Actors with many outgoing friendship ties extend more outgoing ties {outAct} |
| Attribute (Attr.) Alter |  |  | Actor *i* with higher values on an attribute (*v*) attracts more incoming friendship ties {altX} |
| Attr. Ego |  |  | Actor *i* with higher values on an attribute (*v*) extends more outgoing friendship ties {egoX} |
| Same Attr. |  |  | Actor *i* extends friendship ties to alter *j* who has exactly the same values on an attribute {sameX} |
| Similarity Attr. |  |  | Actor *i* extends friendship ties to alter *j* who has similar values on an attribute {simX} |
| **Behavior/Perception Function** |  |  |  |
| Rate of Change |  |  | Average number of opportunities of actors to change behavior or perception (Rate) |
| Linear Shape |  |  | Tendency of actors to change in behavior or perception {linear} |
| Quadratic Shape |  |  | Tendency of actors to change in behavior or perception {quad} |
| Attr. Ego |  |  | Actors with a higher value on an attribute (*v*) change in behavior or perception {effFrom} |
| Average Similarity (Av. Sim.) |  |  | Actor *i* tend towards similar values of behavior or perception (*z*) as his or her friend (*j*) {avSim} |
| Note. Actor (individual) attributes can be constant (e.g., sex) or changing (e.g., defending behavior or perceptions of self-efficacy) over time. Changing behaviors (or perceptions) operate differently in the friendship function than in the behavior (perception) function of the SAOM. In the friendship function of the SAOM, behavior (perception) scores at wave 1 are used to predict changes in friendship ties at wave 2 (behavior or perception is held constant, while friendship is allowed to change). In the behavior (perception) function of the SAOM model, friendship ties at wave 1 are used to predict changes in behavior (perception) at wave 2 (i.e., friendship is held constant, while behavior or perception is allowed to change). | | | |

| **Table S3A**  Relative Importance Scores for All Effects and Effect Classes—For Directed Network Models Predicting Selection and Influence on Defending and Self-Efficacy. | | | | | |
| --- | --- | --- | --- | --- | --- |
|  |  | Effect | | Effect Class | |
|  |  | M1 | M2 | M1 | M2 |
| **Friendship Function** |  |  |  |  |  |
| Effect Class | Effect |  |  |  |  |
| Density & Structural Controls | Outdegree | 19.2% | 19.2% | 79.9% | 79.8% |
|  | Reciprocity | 17.4% | 17.4% |  |  |
|  | Transitive triplets | 16.1% | 16.1% |  |  |
|  | Reciprocated transitive triplets | 11.5% | 11.5% |  |  |
|  | Indegree popularity | 7.5% | 7.5% |  |  |
|  | Outdegree activity | 8.2% | 8.2% |  |  |
| Grouping Controls | Same sex | 4.8% | 4.8% | 7.1% | 7.1% |
|  | Classroom size | 2.3% | 2.3% |  |  |
| Defending Selection | Defending alter | 1.2% | 1.1% | 7.1% | 7.1% |
|  | Defending ego | 1.8% | 1.8% |  |  |
|  | Defending similarity | 4.1% | 4.2% |  |  |
| Self-Efficacy Selection | Self-efficacy alter | 1.3% | 1.3% | 6.0% | 6.0% |
|  | Self-efficacy ego | 1.3% | 1.3% |  |  |
|  | Self-efficacy similarity | 3.4% | 3.4% |  |  |
| **Defending Function** |  |  |  |  |  |
| General Tendencies | Defending: linear shape | 18.7% | 17.8% | 63.7% | 59.3% |
|  | Defending: quadratic shape | 29.4% | 27.0% |  |  |
|  | Defending: indegree | 15.6% | 14.5% |  |  |
| Individual Covariates | Defending: bystanding | 7.0% | 6.8% | 26.8% | 25.2% |
|  | Defending: sex | 4.5% | 4.4% |  |  |
|  | Defending: moral distress | 7.2% | 6.2% |  |  |
|  | Defending: self-efficacy | 8.2% | 7.8% |  |  |
| Friend Influence | Defending: average similarity | 9.5% | 12.1% | 9.5% | 12.1% |
| Classroom Norm | Defending: classroom average |  | 3.4% |  | 3.4% |
| **Self-Efficacy Function** |  |  |  |  |  |
| General Tendencies | Self-efficacy: linear shape | 17.0% | 17.2% | 60.3% | 58.7% |
|  | Self-efficacy: quadratic shape | 28.0% | 26.5% |  |  |
|  | Self-efficacy: indegree | 15.3% | 15.0% |  |  |
| Individual Covariates | Self-efficacy: bystanding | 4.7% | 4.3% | 28.0% | 26.0% |
|  | Self-efficacy: sex | 4.2% | 3.4% |  |  |
|  | Self-efficacy: moral distress | 4.1% | 4.0% |  |  |
|  | Self-efficacy: defending | 15.0% | 14.3% |  |  |
| Friend Influence | Self-efficacy: average similarity | 11.7% | 11.0% | 11.7% | 11.0% |
| Classroom Norm | Self-efficacy: classroom average |  | 4.2% |  | 4.2% |
| Note. Relative importance for each effect (class) in the SAOMs presented in Table 3 (containing defending and self-efficacy), by behavior or perception component. M1 = Main Model, M2 = Classroom Norm Model. Rate of change effects are not included because these do not contribute to the relative importance of effects. Scores for each function in each column sum to 100% (except for cases of rounding error). | | | | | |

| **Table S3B**  Relative Importance Scores for All Effects and Effect Classes—For Directed Network Models Predicting Selection and Influence on Bystanding and Moral Distress. | | | | | |
| --- | --- | --- | --- | --- | --- |
|  |  | Effect | | Effect Class | |
|  |  | M1 | M2 | M1 | M2 |
| **Friendship Function** |  |  |  |  |  |
| Effect Class | Effect |  |  |  |  |
| Density & Structural Controls | Outdegree | 17.5% | 17.5% | 79.0% | 79.1% |
|  | Reciprocity | 18.2% | 18.1% |  |  |
|  | Transitive triplets | 16.3% | 16.3% |  |  |
|  | Reciprocated transitive triplets | 11.7% | 11.7% |  |  |
|  | Indegree popularity | 7.3% | 7.5% |  |  |
|  | Outdegree activity | 8.0% | 8.0% |  |  |
| Grouping Controls | Same sex | 5.1% | 5.0% | 7.3% | 7.1% |
|  | Classroom size | 2.2% | 2.1% |  |  |
| Bystanding Selection | Bystanding alter | 2.9% | 2.9% | 8.3% | 8.3% |
|  | Bystanding ego | 2.0% | 1.9% |  |  |
|  | Bystanding similarity | 3.5% | 3.5% |  |  |
| Moral Distress Selection | Moral distress alter | 2.3% | 2.4% | 5.4% | 5.4% |
|  | Moral distress ego | 0.8% | 0.9% |  |  |
|  | Moral distress similarity | 2.2% | 2.2% |  |  |
| **Bystanding Function** |  |  |  |  |  |
| General Tendencies | Bystanding: linear shape | 20.1% | 20.8% | 54.3% | 55.3% |
|  | Bystanding: quadratic shape | 17.4% | 17.3% |  |  |
|  | Bystanding: indegree | 16.8% | 17.2% |  |  |
| Individual Covariates | Bystanding: defending | 7.6% | 7.1% | 26.9% | 24.9% |
|  | Bystanding: sex | 6.6% | 6.3% |  |  |
|  | Bystanding: self-efficacy | 3.3% | 3.3% |  |  |
|  | Bystanding: moral distress | 9.4% | 8.2% |  |  |
| Friend Influence | Bystanding: average similarity | 18.8% | 16.3% | 18.8% | 16.3% |
| Classroom Norm | Bystanding: classroom average |  | 3.5% |  | 3.5% |
| **Moral Distress Function** |  |  |  |  |  |
| General Tendencies | Moral distress: linear shape | 14.3% | 12.9% | 50.9% | 44.8% |
|  | Moral distress: quadratic shape | 23.7% | 20.1% |  |  |
|  | Moral distress: indegree | 13.0% | 11.7% |  |  |
| Individual Covariates | Moral distress: defending | 5.8% | 5.7% | 26.5% | 26.2% |
|  | Moral distress: sex | 7.4% | 7.2% |  |  |
|  | Moral distress: self-efficacy | 2.9% | 2.8% |  |  |
|  | Moral distress: bystanding | 10.4% | 10.6% |  |  |
| Friend Influence | Moral distress: average similarity | 22.5% | 25.7% | 22.5% | 25.7% |
| Classroom Norm | Moral distress: classroom average |  | 3.3% |  | 3.3% |
| Note. Relative importance for each effect (class) in the SAOMs presented in Table 4 (containing bystanding and moral distress), by behavior or perception component. M1 = Main Model, M2 = Classroom Norm Model. Rate of change effects are not included because these do not contribute to the relative importance of effects. Scores for each function in each column sum to 100% (except for cases of rounding error). | | | | | |

| **Table S4A**  Directed Network Models Predicting Selection and Influence on Defending and Self-Efficacy—Controlling for Grade, with Pooled Data from Schools. | | | | | | |
| --- | --- | --- | --- | --- | --- | --- |
|  | Main Model | | | Classroom Norm Model | | |
| Parameter (statistic) | Est. |  | (SE) | Est. |  | (SE) |
| **Friendship Function** |  |  |  |  |  |  |
| Rate of network change | 11.48 | *** | (0.26) | 11.47 | *** | (0.27) |
| Outdegree | –1.70 | *** | (0.08) | –1.71 | *** | (0.08) |
| Reciprocity | 1.69 | *** | (0.09) | 1.69 | *** | (0.09) |
| Transitive triplets | 0.25 | *** | (0.01) | 0.25 | *** | (0.01) |
| Reciprocated transitive triplets | –0.24 | *** | (0.02) | –0.24 | *** | (0.02) |
| Indegree popularity | –0.05 | *** | (0.01) | –0.05 | *** | (0.01) |
| Outdegree activity | 0.01 | *** | (0.00) | 0.01 | *** | (0.00) |
| Same sex | 0.27 | *** | (0.02) | 0.27 | *** | (0.03) |
| Classroom size | –0.63 | *** | (0.08) | –0.63 | *** | (0.08) |
| Defending alter | –0.05 | † | (0.02) | –0.04 | † | (0.02) |
| Defending ego | –0.02 |  | (0.02) | –0.02 |  | (0.02) |
| Defending similarity | 0.26 | † | (0.16) | 0.26 | † | (0.15) |
| Self-efficacy alter | 0.04 | † | (0.02) | 0.04 | † | (0.02) |
| Self-efficacy ego | 0.00 |  | (0.02) | 0.00 |  | (0.02) |
| Self-efficacy similarity | –0.04 |  | (0.15) | –0.04 |  | (0.16) |
| **Defending Function** |  |  |  |  |  |  |
| Defending: rate of change | 2.16 | *** | (0.14) | 2.14 | *** | (0.15) |
| Defending: linear shape | –0.19 |  | (0.12) | –0.19 |  | (0.12) |
| Defending: quadratic shape | –0.33 | *** | (0.07) | –0.28 | ** | (0.10) |
| Defending: average similarity | 1.24 | † | (0.74) | 1.73 |  | (1.15) |
| Defending: indegree | 0.01 |  | (0.01) | 0.00 |  | (0.01) |
| Defending: sex (ref. = girl) | –0.16 | * | (0.08) | –0.17 | * | (0.08) |
| Defending: grade | 0.02 |  | (0.04) | 0.00 |  | (0.04) |
| Defending: self-efficacy | 0.16 | * | (0.06) | 0.16 | ** | (0.06) |
| Defending: bystanding | –0.21 | *** | (0.05) | –0.22 | *** | (0.05) |
| Defending: moral distress | 0.14 | ** | (0.04) | 0.14 | ** | (0.05) |
| Defending: classroom average^b^ |  |  |  | –0.16 |  | (0.20) |
| **Self-Efficacy Function** |  |  |  |  |  |  |
| Self-efficacy: rate of change | 2.47 | *** | (0.15) | 2.44 | *** | (0.15) |
| Self-efficacy: linear shape | –0.03 |  | (0.10) | –0.05 |  | (0.10) |
| Self-efficacy: quadratic shape | –0.20 | *** | (0.05) | –0.16 | * | (0.06) |
| Self-efficacy: average similarity | 2.12 | ** | (0.82) | 2.79 | * | (1.09) |
| Self-efficacy: indegree | –0.00 |  | (0.01) | 0.00 |  | (0.01) |
| Self-efficacy: sex (ref. = girl) | –0.09 |  | (0.07) | –0.11 |  | (0.07) |
| Self-efficacy: grade | 0.06 | * | (0.03) | 0.06 | * | (0.03) |
| Self-efficacy: defending | 0.25 | *** | (0.06) | 0.26 | *** | (0.06) |
| Self-efficacy: bystanding | –0.06 |  | (0.04) | –0.06 |  | (0.04) |
| Self-efficacy: moral distress | –0.02 |  | (0.04) | –0.02 |  | (0.04) |
| Self-efficacy: classroom average^b^ |  |  |  | –0.18 |  | (0.15) |
| Note. See Table 3. | | | | | | |

| **Table S4B**  Directed Network Models Predicting Selection and Influence on Bystanding and Moral Distress—Controlling for Grade, with Pooled Data from Schools. | | | | | | |
| --- | --- | --- | --- | --- | --- | --- |
|  | Main Model | | | Classroom Norm Model | | |
| Parameter (statistic) | Est. |  | (SE) | Est. |  | (SE) |
| **Friendship Function** |  |  |  |  |  |  |
| Rate of network change | 11.41 | *** | (0.26) | 11.41 | *** | (0.26) |
| Outdegree | –1.68 | *** | (0.07) | –1.68 | *** | (0.08) |
| Reciprocity | 1.67 | *** | (0.08) | 1.67 | *** | (0.09) |
| Transitive triplets | 0.25 | *** | (0.01) | 0.25 | *** | (0.01) |
| Reciprocated transitive triplets | –0.23 | *** | (0.01) | –0.23 | *** | (0.02) |
| Indegree popularity | –0.05 | *** | (0.01) | –0.05 | *** | (0.01) |
| Outdegree activity | 0.01 | *** | (0.00) | 0.01 | *** | (0.00) |
| Same sex | 0.28 | *** | (0.03) | 0.28 | *** | (0.03) |
| Classroom size | –0.61 | *** | (0.08) | –0.60 | *** | (0.08) |
| Bystanding alter | 0.13 | *** | (0.03) | 0.13 | *** | (0.03) |
| Bystanding ego | –0.01 |  | (0.02) | –0.01 |  | (0.02) |
| Bystanding similarity | 0.16 |  | (0.17) | 0.17 |  | (0.18) |
| Moral distress alter | –0.03 | † | (0.02) | –0.03 |  | (0.02) |
| Moral distress ego | –0.03 |  | (0.02) | –0.03 |  | (0.02) |
| Moral distress similarity | –0.28 | * | (0.12) | –0.28 | * | (0.12) |
| **Bystanding Function** |  |  |  |  |  |  |
| Bystanding: rate of change | 2.18 | *** | (0.16) | 2.15 | *** | (0.15) |
| Bystanding: linear shape | 0.06 |  | (0.12) | 0.07 |  | (0.12) |
| Bystanding: quadratic shape | –0.16 | * | (0.07) | –0.11 |  | (0.08) |
| Bystanding: average similarity | 3.39 | *** | (0.72) | 3.91 | *** | (0.92) |
| Bystanding: indegree | –0.01 |  | (0.01) | –0.01 |  | (0.01) |
| Bystanding: sex (ref. = girl) | 0.14 | † | (0.08) | 0.15 | † | (0.08) |
| Bystanding: grade | –0.03 |  | (0.03) | –0.03 |  | (0.03) |
| Bystanding: moral distress | –0.02 |  | (0.06) | –0.02 |  | (0.06) |
| Bystanding: defending | –0.19 | *** | (0.05) | –0.19 | *** | (0.05) |
| Bystanding: self-efficacy | –0.05 |  | (0.04) | –0.05 |  | (0.04) |
| Bystanding: classroom average^b^ |  |  |  | –0.26 |  | (0.22) |
| **Moral Distress Function** |  |  |  |  |  |  |
| Moral distress: rate of change | 1.58 | *** | (0.10) | 1.56 | *** | (0.10) |
| Moral distress: linear shape | –0.09 |  | (0.16) | –0.08 |  | (0.16) |
| Moral distress: quadratic shape | –0.04 |  | (0.05) | 0.07 |  | (0.10) |
| Moral distress: average similarity | 6.03 | *** | (0.63) | 7.01 | *** | (1.06) |
| Moral distress: indegree | 0.01 |  | (0.01) | 0.00 |  | (0.01) |
| Moral distress: sex (ref. = girl) | –0.32 | *** | (0.11) | –0.31 | ** | (0.11) |
| Moral distress: grade | 0.03 |  | (0.04) | 0.01 |  | (0.05) |
| Moral distress: bystanding | 0.05 |  | (0.11) | 0.06 |  | (0.11) |
| Moral distress: defending | 0.18 | ** | (0.07) | 0.18 | ** | (0.07) |
| Moral distress: self-efficacy | 0.07 |  | (0.06) | 0.07 |  | (0.06) |
| Moral distress: classroom average^b^ |  |  |  | –0.27 |  | (0.21) |
| Note. See Table 3. | | | | | | |

| **Table S5A**  Directed Network Models Predicting Selection and Influence on Defending and Self-Efficacy—Restricting Influence to Reciprocal Friends. | | | | | | | | |
| --- | --- | --- | --- | --- | --- | --- | --- | --- |
|  | Main Model | | | | Classroom Norm Model | | | |
| Parameter (statistic) | Est. |  | (SE) | *n* | Est. |  | (SE) | *n* |
| **Friendship Function** |  |  |  |  |  |  |  |  |
| Rate of network change | 10.87 | *** | (1.65)^a^ | 9 | 10.88 | *** | (1.67)^a^ | 9 |
| Outdegree | –2.07 | *** | (0.16)^a^ | 9 | –2.05 | *** | (0.16) | 9 |
| Reciprocity | 1.95 | *** | (0.40)^a^ | 9 | 1.94 | *** | (0.38)^a^ | 9 |
| Transitive triplets | 0.28 | ** | (0.06)^a^ | 9 | 0.27 | *** | (0.06)^a^ | 9 |
| Reciprocated transitive triplets | –0.30 | ** | (0.07)^a^ | 9 | –0.29 | *** | (0.07)^a^ | 9 |
| Indegree popularity | –0.03 |  | (0.03)^a^ | 9 | –0.04 |  | (0.03)^a^ | 9 |
| Outdegree activity | 0.02 |  | (0.01)^a^ | 9 | 0.02 |  | (0.02)^a^ | 9 |
| Same sex | 0.35 | *** | (0.05) | 9 | 0.35 | *** | (0.05) | 9 |
| Classroom size | –0.94 | † | (0.47)^a^ | 9 | –0.94 | † | (0.48)^a^ | 9 |
| Defending alter | –0.02 |  | (0.02) | 9 | –0.02 |  | (0.02) | 9 |
| Defending ego | –0.02 |  | (0.06)^a^ | 9 | –0.03 |  | (0.06)^a^ | 9 |
| Defending similarity X  reciprocity | 0.21 |  | (0.31) | 8 | 0.33 |  | (0.30) | 9 |
| Self-efficacy alter | 0.00 |  | (0.02) | 9 | 0.00 |  | (0.02) | 9 |
| Self-efficacy ego | –0.01 |  | (0.03) | 9 | –0.01 |  | (0.03) | 9 |
| Self-efficacy similarity X reciprocity | 0.15 |  | (0.37) | 8 | 0.16 |  | (0.19) | 9 |
| **Defending Function** |  |  |  |  |  |  |  |  |
| Defending: rate of change | 1.89 | *** | (0.12) | 9 | 1.89 | *** | (0.12) | 9 |
| Defending: linear shape | –0.22 | † | (0.11) | 9 | –0.21 |  | (0.19) | 9 |
| Defending: quadratic shape | –0.31 | *** | (0.06) | 9 | –0.28 | ** | (0.07) | 9 |
| Defending: average similarity X reciprocity | 1.80 | † | (0.79) | 8 | 2.15 | † | (0.78) | 8 |
| Defending: indegree | 0.02 |  | (0.01) | 9 | 0.01 |  | (0.02) | 9 |
| Defending: sex (ref. = girl) | –0.22 | ** | (0.06) | 9 | –0.14 | † | (0.09) | 9 |
| Defending: self-efficacy | 0.14 | * | (0.06) | 9 | 0.13 | † | (0.05) | 9 |
| Defending: bystanding | –0.15 | † | (0.07) | 9 | –0.22 | ** | (0.07) | 9 |
| Defending: moral distress | 0.23 | * | (0.07) | 9 | 0.22 | ** | (0.06) | 9 |
| Defending: classroom average^b^ |  |  |  |  |  |  | (0.18) | 9 |
| **Self-Efficacy Function** |  |  |  |  |  |  |  |  |
| Self-efficacy: rate of change | 2.20 | *** | (0.21) | 9 | 2.18 | *** | (0.21) | 9 |
| Self-efficacy: linear shape | –0.12 | * | (0.05) | 9 | –0.13 | * | (0.11) | 9 |
| Self-efficacy: quadratic shape | –0.25 | *** | (0.05) | 9 | –0.24 | ** | (0.07) | 9 |
| Self-efficacy: average similarity X reciprocity | 1.91 | † | (1.02) | 9 | 2.19 |  | (1.12) | 9 |
| Self-efficacy: indegree | 0.01 |  | (0.01) | 9 | 0.01 |  | (0.01) | 9 |
| Self-efficacy: sex (ref. = girl) | –0.07 |  | (0.06) | 9 | –0.15 | * | (0.06) | 9 |
| Self-efficacy: defending | 0.20 | ** | (0.06) | 9 | 0.21 | * | (0.07) | 9 |
| Self-efficacy: bystanding | –0.15 | † | (0.08) | 9 | –0.08 |  | (0.07) | 9 |
| Self-efficacy: moral distress | 0.01 |  | (0.04) | 9 | 0.00 |  | (0.05) | 9 |
| Self-efficacy: classroom average^b^ |  |  |  |  | –0.15 |  | (0.21) | 9 |
| Note. See Table 3. | | | | | | | | |

| **Table S5B**  Directed Network Models Predicting Selection and Influence on Bystanding and Moral Distress—Restricting Influence to Reciprocal Friends. | | | | | | | | |
| --- | --- | --- | --- | --- | --- | --- | --- | --- |
|  | Main Model | | | | Classroom Norm Model | | | |
| Parameter (statistic) | Est. |  | (SE) | *n* | Est. |  | (SE) | *n* |
| **Friendship Function** |  |  |  |  |  |  |  |  |
| Rate of network change | 10.83 | *** | (1.59)^a^ | 9 | 10.82 | *** | (1.59)^a^ | 9 |
| Outdegree | –2.01 | *** | (0.17)^a^ | 9 | –2.01 | *** | (0.17)^a^ | 9 |
| Reciprocity | 1.98 | *** | (0.37)^a^ | 9 | 1.99 | *** | (0.37)^a^ | 9 |
| Transitive triplets | 0.27 | ** | (0.06)^a^ | 9 | 0.28 | ** | (0.06)^a^ | 9 |
| Reciprocated transitive triplets | –0.29 | ** | (0.07)^a^ | 9 | –0.29 | ** | (0.07)^a^ | 9 |
| Indegree popularity | –0.04 |  | (0.03)^a^ | 9 | –0.04 |  | (0.03)^a^ | 9 |
| Outdegree activity | 0.02 |  | (0.01)^a^ | 9 | 0.02 |  | (0.01)^a^ | 9 |
| Same sex | 0.37 | *** | (0.06)^a^ | 9 | 0.38 | *** | (0.06)^a^ | 9 |
| Classroom size | –0.94 | † | (0.47)^a^ | 9 | –0.90 | † | (0.45)^a^ | 9 |
| Bystanding alter | 0.11 | † | (0.06)^a^ | 9 | 0.12 | † | (0.06)^a^ | 9 |
| Bystanding ego | –0.01 |  | (0.07) | 9 | –0.01 |  | (0.06)^a^ | 9 |
| Bystanding similarity X reciprocity | –0.13 |  | (0.18)^a^ | 7 | –0.20 |  | (0.19) | 7 |
| Moral distress alter | –0.03 |  | (0.05) | 9 | –0.03 |  | (0.05) | 9 |
| Moral distress ego | –0.02 |  | (0.04) | 9 | –0.02 |  | (0.04) | 9 |
| Moral distress similarity X reciprocity | –0.05 |  | (0.17) | 7 | –0.06 |  | (0.16) | 7 |
| **Bystanding Function** |  |  |  |  |  |  |  |  |
| Bystanding: rate of change | 2.01 | *** | (0.21) | 9 | 1.97 | *** | (0.21) | 9 |
| Bystanding: linear shape | –0.06 |  | (0.14) | 9 | –0.03 |  | (0.14) | 9 |
| Bystanding: quadratic shape | –0.24 | ** | (0.07) | 9 | –0.23 | * | (0.07) | 9 |
| Bystanding: average similarity X reciprocity | 2.28 | * | (0.68) | 8 | 2.50 | ** | (0.64) | 8 |
| Bystanding: reciprocal degree | 0.01 |  | (0.02) | 9 | 0.01 |  | (0.02) | 9 |
| Bystanding: sex (ref. = girl) | 0.07 |  | (0.10) | 9 | 0.04 |  | (0.10) | 9 |
| Bystanding: moral distress | –0.06 |  | (0.11) | 9 | –0.07 |  | (0.10) | 9 |
| Bystanding: defending | –0.19 | * | (0.06) | 9 | –0.18 | * | (0.07) | 9 |
| Bystanding: self-efficacy | –0.05 |  | (0.05) | 9 | –0.05 |  | (0.05) | 9 |
| Bystanding: classroom average^b^ |  |  |  |  | –0.04 |  | (0.22) | 9 |
| **Moral Distress Function** |  |  |  |  |  |  |  |  |
| Moral distress: rate of change | 1.70 | *** | (0.11) | 9 | 1.70 | *** | (0.11) | 9 |
| Moral distress: linear shape | 0.06 |  | (0.16) | 9 | 0.07 |  | (0.15) | 9 |
| Moral distress: quadratic shape | –0.35 | ** | (0.08) | 9 | –0.30 | ** | (0.08) | 9 |
| Moral distress: average similarity X reciprocity | 3.55 | † | (1.61) | 7 | 4.00 | † | (1.68) | 7 |
| Moral distress: reciprocal degree | –0.00 |  | (0.02) | 9 | –0.00 |  | (0.02) | 9 |
| Moral distress: sex (ref. = girl) | –0.38 |  | (0.15) | 9 | –0.40 | * | (0.15) | 9 |
| Moral distress: bystanding | –0.06 |  | (0.20) | 9 | –0.15 |  | (0.18) | 9 |
| Moral distress: defending | 0.15 | † | (0.08) | 9 | 0.15 | † | (0.07) | 9 |
| Moral distress: self-efficacy | 0.11 | * | (0.03) | 9 | 0.11 | * | (0.04) | 9 |
| Moral distress: classroom average^b^ |  |  |  |  | –0.41 | † | (0.20) | 8 |
| Note. See Table 3. | | | | | | | | |

**References**

Faust, K. (2007). Very local structure in social networks. *Sociological Methodology*, *37*, 209-256. doi:10.1111/j.1467-9531.2007.00179.x

Ripley, R. M., Snijders, T. A. B., Boda, Z., Vörös, A., & Preciado, P. (2021). *Manual for RSiena*. Department of Statistics, Nuffield College. http://www.stats.ox.ac.uk/~snijders/siena/RSiena_Manual.pdf

Steglich, C. E. G., Snijders, T. A. B., & Pearson, M. (2010). Dynamic networks and behavior: Separating selection from influence. *Sociological Methodology*, *40*, 329–393. doi:10.1111/j.1467-9531.2010.01225.x
